# Management of Traumatic Injury to Maxillary Central Incisors associated with Inverted Mesiodens: A Case Report

**DOI:** 10.5005/jp-journals-10005-1182

**Published:** 2013-04-26

**Authors:** Chaitanya Pavuluri, Sivakumar Nuvvula

**Affiliations:** Senior Lecturer, Department of Pedodontics and Preventive Dentistry Drs Sudha & Nageswara Rao Siddhartha Institute of Dental Sciences Gannavaram, Andhra Pradesh, India; Professor and Head, Department of Pedodontics and Preventive Dentistry, Narayana Dental College and Hospital, Nellore, Andhra Pradesh, India

**Keywords:** Apexification, Maxillary central incisor, Inverted supernumerary tooth, Mineral trioxide aggregate

## Abstract

Maxillary incisors are the most frequently injured teeth in the primary and permanent dentition. Stage of adolescence show a significant number of dental injuries as they engage in contact sports. Children with accident prone profile, i.e. class II division I or class I type II malocclusion are more prone for injuries because of the proclined maxillary incisors. Supernumerary teeth are those that are additional to the normal complement. They occur in single or multiple, unilateral or bilateral in either of the jaws. This paper reports the presence of an inverted supernumerary tooth in the right maxillary central incisor region with trauma involving both maxillary central incisors and also the management of the supernumerary tooth and traumatized teeth in a 14-year-old boy.

**How to cite this article:** Pavuluri C, Nuvvula S. Management of Traumatic Injury to Maxillary Central Incisors associated with Inverted Mesiodens: A Case Report. Int J Clin Pediatr Dent 2013;6(1):30-32.

## INTRODUCTION

Maxillary incisors are the most frequently injured teeth in the primary and permanent dentition. Injury to the young permanent teeth is a disturbing experience for the child and parents because of their location, esthetic and psychological/ emotional importance. Injury to the teeth can cause long-term consequences leading to their discoloration and malformation. Pinkham has emphasized on taking good history of the event for proper diagnosis and treatment.^1^

## CASE REPORT

A South Indian boy aged 14 years, reported to the Department of Pedodontics and Preventive Dentistry, accompanied by his father with the chief complaint of broken upper front teeth. History revealed trauma 1 month before while playing and there was no loss of consciousness at the time of injury. There was history of bleeding from the teeth after the injury with sensitivity on taking hot and cold substance and pus discharge through the broken incisors 2 weeks later. Health history of the patient was not contributory.

**Fig. 1:**
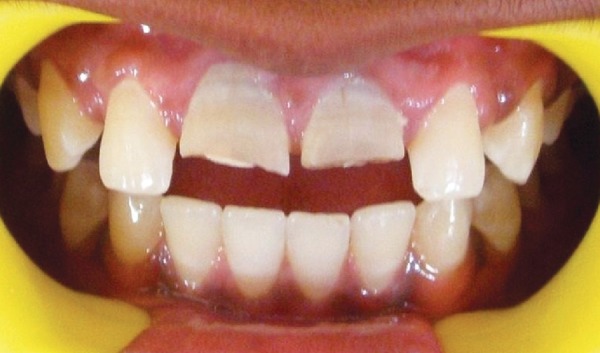
Fracture involving permanent maxillary central incisors

Patient showed bilaterally symmetrical face with competent lips. Intraoral examination revealed permanent dentition with fracture of both maxillary permanent central incisors (Fig. 1) and pus discharge through the open pulp chambers of both centrals. Bilateral class I molar relationship was noted with localized gingivitis in relation to permanent first molars.

**Fig. 2:**
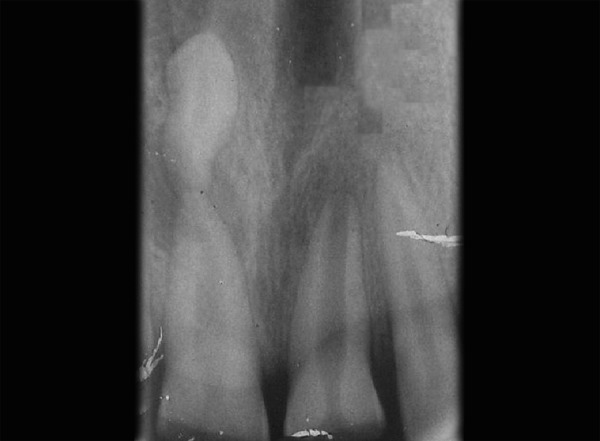
Periapical radiograph revealing inverted mesiodens apical to 11

Provisional diagnosis of Ellis class IV injury was made as involving maxillary permanent central incisors. Intraoral periapical radiograph taken for maxillary central incisor area showed periapical radiolucency in relation to central incisors, presence of an inverted supernumerary tooth in the periapical area of the right central incisor and the presence of blunderbuss canal for left central incisor (Fig. 2). The final diagnosis was Ellis class IV injury in maxillary permanent centrals and inverted supernumerary tooth in relation to maxillary right central incisor.

**Fig. 3:**
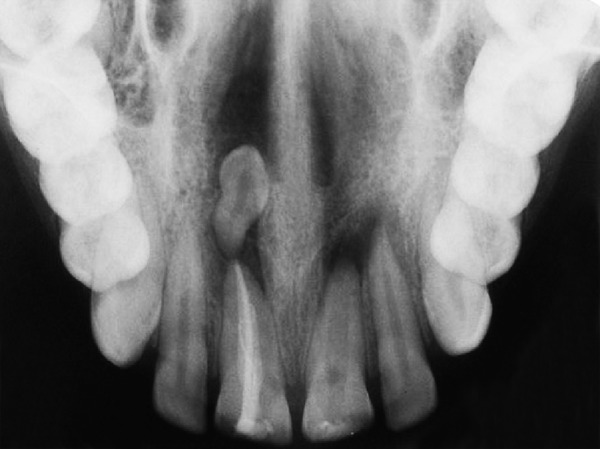
Occlusal radiograph revealing endodontically treated 11

**Fig. 4:**
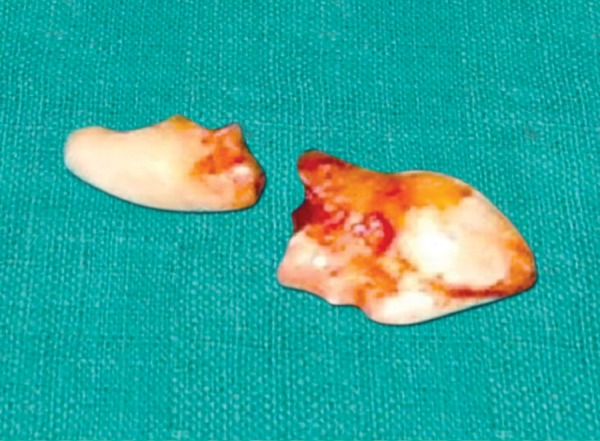
Extracted supernumerary tooth

**Fig. 5:**
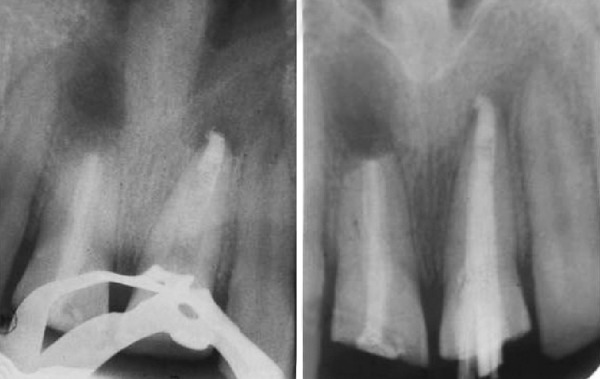
Periapical radiograph revealing apexification with MTA and endodontic treatment of 21

**Fig. 6:**
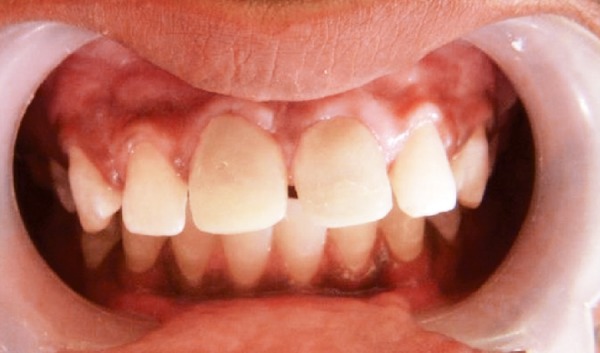
Composite restoration in relation to 11 and 21

Treatment planning included, completion of root canal treatment in maxillary right central incisor (Fig. 3) followed by extraction of the inverted supernumerary tooth under local anesthesia and apexification for maxillary left central incisor. Palatal crevicular incision was placed from maxillary right canine to maxillary left canine and mucoperiosteal flap was raised exposing the bulge of inverted supernumerary tooth present at the apex of maxillary right central incisor. Bone is removed until the crown of the supernumerary tooth is exposed. The crown of the supernumerary tooth was sectioned with a fissure bur and removed as first piece and the root is elevated into the space vacated by the crown and removed (Fig. 4). A portion of the root apex of the maxillary right central incisor had to be sacrificed during the procedure as it was closely related to the root of the supernumerary tooth. After careful debridement, which included complete removal of the remnants, and achieving hemostasis the flap is repositioned and sutured interdentally. Apexification was planned for maxillary left central incisor with Mineral Trioxide Aggregate (MTA, Angelus®, Brazil). Maxillary left central incisor was isolated using rubber dam and working length was determined about 16 mm. MTA was mixed in the ratio of 1:1 on a sterile glass slab, placed into the canal and condensed using reverse ends of the paper points. Intraoral Periapical (IOPA) radiograph was taken after appropriate amount of MTA was condensed into the canal (Fig. 5). After 15 minutes interval obturation using gutta-percha points was completed in maxillary left central incisor. After 3 weeks of apexification composite build-up was done (Fig. 6).

## DISCUSSION

Ellis class IV fracture is defined as ‘the traumatized teeth that become nonvital with or without loss of crown structure'. The maxillary incisors are the most frequently injured teeth in the primary and permanent dentition. Teenage years cause a significant number of dental injuries as they engage in contact sports. Children with accident prone profile, i.e. class II division I or class I type II malocclusion are more prone for injuries because of the proclined incisors.^2^ Supernumerary teeth are those that are additional to the normal complement. Primosh^3,4^ 1981 classified supernumerary teeth into two types according to their shape as supplemental and rudimentary*.* Its orientation can be described as vertical, inverted and transverse as given by Gregg and Kinirons,^3,5^ 1991.

Supernumerary teeth are a developmental disturbance during odontogenesis process. The term mesiodens refers to a supernumerary tooth that is present between the two central incisors. Mesiodens are most common in permanent dentition than in primary. The first report of a supernumerary tooth appeared between AD 23 and 79.^6,7^ Prevalence of supernumerary teeth is 0 to 1.9% for primary teeth and between 0.5 and 3.8% for permanent teeth.^6,8^

Traumatic injuries to the young permanent teeth are common and affect about 30% of the children.^6^ Majority of the traumatic injuries occurs before root formation and results in inflammation and necrosis. Many techniques have been suggested for induction of apical closure in pulpless teeth to produce favorable conditions for conventional root canal filling.^9^

Most of these techniques involve removal of the necrotic tissue followed by debridement of the canal and placement of a medicament. Although calcium hydroxide has been the material of choice for apexification, in recent years interest has been centered on the use of MTA for apexification. MTA was introduced in 1993 and approved by FDA in 1998.^9^ Morse et al defined one visit apexification as the nonsurgical condensation of a biocompatible material into the apical end of the root canal.^9,12^ Witherspoon and Ham stated that MTA provides scaffolding for the formation of hard tissue and the potential of a better biological seal.^9,10^ MTA was described for the first time in the dental literature by Lee et al 1993.^11^ MTA has been demonstrated to have diverse applications for all fields of dentistry, indicated for direct pulp capping, repair of internal resorption, root end filling, apexification, repair of root perforations and pulpotomy.^12^ MTA has better marginal adaptation to the root end cavity.^11,12^ The main advantage of MTA is that apical seal can be achieved in a single visit. MTA powder consists of fine hydrophilic particles, the principal compounds of which are tricalcium silicate, dicalcium silicate, tetracalcium aluminoferrite, calcium sulfate dehydrate and tricalcium aluminate. Bismuth oxide is present to make the MTA radiopaque and resistance to the penetration of the microorganisms is higher with MTA.^13^

## CONCLUSION

Trauma to the young permanent teeth should be considered as an emergency treatment. A good prognosis depends on the multidisciplinary team approach for the diagnosis and treatment planning by the pediatric dentist.
